# The Human Face of Digitalization in Healthcare: A Mixed-Methods Study on the Relationship Between Emotional Intelligence and Burnout

**DOI:** 10.3390/bs15111505

**Published:** 2025-11-06

**Authors:** Rana Özyurt Kaptanoğlu

**Affiliations:** Department of Management Information Systems, Faculty of Economics, Administrative and Social Sciences, Istanbul Topkapi University, Istanbul 34662, Türkiye; ranaozyurt@topkapi.edu.tr; Tel.: +90-532-793-0969

**Keywords:** digital transformation, emotional intelligence, burnout, organizational behavior, bibliometric data analysis

## Abstract

Technological developments have profoundly transformed healthcare, compelling institutions to adapt rapidly to digital transformation. This shift has increased job pressure and uncertainty, thereby heightening the risk of burnout. Emotional intelligence (EI), as an individual capacity, plays a critical role in coping with these demands. This study examines the relationship between EI and burnout during the digital transformation process using a mixed-methods design. Specifically, it combines (1) a bibliometric analysis of 540 studies on EI and burnout (2004–2025) retrieved from the Web of Science database to identify conceptual gaps, and (2) a survey of 590 employees from four public hospitals at advanced stages of digital transformation (HIMSS Stages 6–7), analyzed with SPSS 23.0. The results reveal a significant negative correlation between EI and burnout, indicating that employees with higher EI experience lower burnout levels. Regression analyses and effect size estimates (standardized β coefficients and R^2^ values) further support the robustness of this relationship. Taken together, the bibliometric and empirical findings indicate that the intersection of EI and burnout constitutes an emerging yet underexplored field, particularly in digital contexts. This study contributes to the literature by integrating theoretical insights and empirical evidence to provide a comprehensive understanding of how digital transformation influences healthcare professionals’ psychological well-being through the interplay of EI and burnout.

## 1. Introduction

Digital transformation has exerted a profound impact on the healthcare sector as a comprehensive wave reshaping all business processes. This transformation, which requires extensive use of digital interfaces and equipment, has led to employee resistance due to outcomes such as insufficient user knowledge, mental fatigue, and loss of control. In fact, digital transformation in healthcare is often perceived by employees as a form of “digital compulsion,” and the abandonment of traditional work routines brings about individual-level adaptation challenges (Bawack et al., 2021).

Within this process, individuals’ psychosocial adaptation capacity is a significant determinant (Schuler & Jackson, 1987). Emotional intelligence (EI), in particular, plays a critical role in adapting to change through its associations with managing interpersonal relationships (Lopes et al., 2003; Schutte et al., 2013), coping with stress (Salovey & Mayer, 1990; Van Deursen et al., 2015), maintaining motivation (Magnano et al., 2016; Trigueros et al., 2019), and demonstrating empathy (Abe et al., 2018; Goleman, 1998). However, digitalization brings additional job demands such as increased workload (Tarafdar et al., 2019), difficulty in technical learning (Portoghese et al., 2014), and time pressure (Teng et al., 2010), all of which raise the risk of burnout among employees (X. Cao & Naruse, 2019; Üler, 2020). In healthcare specifically, burnout is regarded as a long-term outcome that threatens both individual well-being and the quality of care delivered (Dyrbye et al., 2017; Profit et al., 2021).

This study examines the relationship between EI and burnout during the digital transformation process within the framework of the Job Demands–Resources (JD-R) model. According to the model, when organizational demands imposed on individuals are not balanced with institutional and personal resources, negative outcomes such as burnout may occur (Bakker & Demerouti, 2007; Xanthopoulou et al., 2007). In this study, “digital transformation” is modeled as a job demand, “emotional intelligence” as a personal resource, and “burnout” as the outcome variable. This theoretical perspective provides a meaningful basis for explaining the effects of digital transformation-related challenges on employees and for developing preventive strategies (Bakker & Demerouti, 2017).

Although the key concepts of burnout, emotional intelligence, and digital transformation have frequently been addressed in the literature, studies that explore their interactions remain limited. Digital transformation has often been examined in the context of institutional infrastructure changes (Vial, 2019) or technological adaptation processes (Agarwal et al., 2010), while emotional intelligence has typically been studied in relation to individuals’ communication competencies (Mayer et al., 2004), job satisfaction (Harms & Credé, 2010), and leadership skills (George, 2000). In burnout research, factors such as stress (Schutte et al., 2013; Uludağ et al., 2024), motivation (Trigueros et al., 2019), and workload (Tarafdar et al., 2019) have been emphasized, whereas the role of digitalization has mostly been treated as secondary. Particularly in a labor-intensive sector like healthcare, research investigating how these concepts are interrelated remains scarce.

To address this gap, the present study combines applied analysis with a bibliometric mapping of the relationships among these concepts. A search of the Web of Science (WoS) database using “title,” “topic,” and “all fields” criteria revealed no studies addressing all three concepts together. Therefore, the WoS analysis focused on studies involving emotional intelligence and burnout. Based on 540 studies published between 2004 and 2025, the analysis showed that these concepts appeared mostly in independent clusters, that the number of studies relating them was limited, and that scholarly interest was concentrated in specific years. Interest in these concepts increased after the 2000s as digitalization gained momentum. However, the continued scarcity of studies specifically focused on healthcare highlights the originality and significance of the current research.

The existing literature indicates that studies comprehensively examining the impact of digital transformation on healthcare workers’ levels of burnout and emotional intelligence are very limited, and that no research has addressed this relationship within a theoretical framework such as the Job Demands–Resources (JD-R) model. To address this gap, the present study investigates the relationship between emotional intelligence and burnout during the digital transformation process within the JD-R model, combining bibliometric and empirical data to provide an original contribution to the literature.

Accordingly, the study aims to examine the relationship between emotional intelligence and burnout among healthcare workers undergoing digital transformation within the JD-R framework. Specifically, it seeks to:

(i) identify the conceptual gap in the literature through a bibliometric analysis,
(ii) empirically examine the association between EI and burnout in hospitals at HIMSS Stages 6–7, and
(iii) explore the potential moderating role of digital transformation as a job demand.

HIMSS Stages 6–7 refer to the advanced levels of the HIMSS Electronic Medical Record Adoption Model (EMRAM), a maturity framework that assesses and guides the progressive adoption and optimization of electronic medical records and digital health systems in hospitals.

This study makes a distinct contribution to the literature by examining the relationship between emotional intelligence and burnout within the context of digital transformation in healthcare. Although the link between emotional intelligence and burnout has been explored in various settings, little research has investigated how this relationship unfolds in healthcare, where digital transformation intensifies workload, role ambiguity, and technostress factors. By combining bibliometric analysis and empirical data, the present study offers a comprehensive framework for understanding how digital transformation influences healthcare professionals’ emotional intelligence and burnout levels, while also underscoring the importance of EI training and support systems in mitigating burnout during digital transformation processes.

## 2. Conceptual Framework

The core concepts addressed in this study—burnout, emotional intelligence, and digital transformation—are crucial for understanding the psychosocial dimensions of employees at both individual and organizational levels. Emotional intelligence, often associated with empathy and adaptability, and burnout, typically linked to stress and heavy workloads among healthcare professionals, are examined here in conjunction with the new job demands arising from the digital transformation process. Within the JD-R framework, these concepts are treated as complementary elements, and their treatment in the literature is summarized accordingly.

### 2.1. Emotional Intelligence Among Healthcare Workers During the Digital Transformation Process

It has long been recognized that cognitive intelligence alone is insufficient for understanding how individuals respond to life events or cope with challenges. With growing awareness of the role emotions play in human functioning, the concept of emotional intelligence has emerged and gained considerable attention in academic circles over the past two decades (Mayer et al., 2004). Originating from the broader notion of social intelligence, emotional intelligence was first described by Thorndike (1920) as “the ability to understand others and to act wisely in relationships.” Building on this foundation, Goleman (2001a, 2001b, 2005) conceptualized emotional intelligence as the ability to motivate oneself, persist in the face of setbacks, regulate one’s emotions, and empathize with others by understanding their feelings.

Research has demonstrated that cognitive intelligence alone is not sufficient for success in life; emotional intelligence is equally important. In this context, these two forms of intelligence should be regarded not as alternatives but as complementary capacities, both of which are necessary for a successful life (Petrides & Furnham, 2001). Over recent years, it has become increasingly evident that individuals differ in their ability to perceive, use, and understand emotional information, and this variability has been explained within the framework of emotional intelligence (Salovey & Mayer, 1990; Chan, 2006). Emotional intelligence can be defined as an individual’s capacity to be aware of, comprehend, and effectively use both their own emotions and those of others in social interactions (McCornel, 2003). The concept emerges from the interplay of emotion and cognition, emphasizing the guiding role of emotions in human behavior. Recent studies have shown that emotional processing abilities—such as emotion recognition and emotion understanding—are distinct components of emotional intelligence and significantly predict individual well-being and decision-making outcomes (Gillioz et al., 2023).

In light of these definitions, emotional intelligence can be understood as a set of individual and social competencies that shape one’s success in life (Goleman, 1995). These competencies include recognizing and identifying one’s own emotions (Salovey & Mayer, 1990), regulating them appropriately (Bar-On, 1997), and maintaining self-motivation to pursue life goals (Goleman, 2001b), as well as perceiving the emotions of others (Mayer et al., 2004), demonstrating empathy by putting oneself in their position (Davis, 1983), and engaging effectively in interpersonal interactions (Riggio, 1986). A recent meta-analysis conducted in workplace settings indicates that emotional competencies—particularly emotion regulation and empathy—can be significantly enhanced through intervention programs, which in turn improve psychological well-being and interpersonal performance (Mehler et al., 2024).

Emotional intelligence (EI) is increasingly recognized as a critical competency for healthcare professionals, enhancing patient care, team collaboration, and decision-making processes (Koutsioumpa, 2023; Sahney et al., 2024). EI encompasses self-awareness, self-management, social awareness, and relationship management, enabling healthcare workers to navigate stressful situations and build rapport with both patients and colleagues. The COVID-19 pandemic has underscored the importance of EI in fostering resilience and effective communication among healthcare personnel (Sahney et al., 2024). Research further indicates that healthcare leaders with strong EI competencies are better equipped to manage team dynamics and promote optimal performance in high-pressure settings. To develop EI, strategies such as simulation training, reflective practice, and mentorship programs are increasingly being implemented. Integrating EI training into medical education and ongoing professional development is now regarded as essential for improving healthcare outcomes (Koutsioumpa, 2023).

In the digital age, EI plays a pivotal role in distinguishing humans from machines and preserving human uniqueness in increasingly automated environments. Organizations are prioritizing EI over IQ in recruitment processes and recognizing its importance in fostering positive workplace cultures (Goleman, 1995; O’Boyle et al., 2011). Moreover, the convergence of artificial intelligence (AI) and EI is emerging as both a significant challenge and a promising opportunity in digital workspaces (Kaur & Sharma, 2021). Valtonen and Holopainen (2025) found that, although AI adoption does not directly reduce employee well-being, its effects mediated by EI-related demands—such as emotional labor and role overload—are substantial, indicating that emotional intelligence becomes even more essential during digital transformation.

Adaptability in the digitalization process has become closely linked to emotional intelligence (EI), and it is widely accepted that individuals with high EI experience fewer difficulties in this regard. In a recent study, Nag et al. (2024) examined how EI supports patient connection, stress management, and interaction with digital systems in healthcare environments. Their findings suggest that AI may serve as a bridge between technology and human interaction. Among healthcare workers, high EI contributes to both psychological resilience and improved stress management (Sahney et al., 2024). EI has also been shown to enhance team performance, promote psychological safety, and improve patient outcomes in digital healthcare applications (https://healthmanagement.org). In a study involving a team of healthcare professionals providing digital consultation services, emotional intelligence played a significant role in strengthening the patient–provider relationship, fostering empathy, and mitigating the sense of distance created by digital interactions (Litendahl et al., 2025).

### 2.2. Burnout Among Healthcare Workers During the Digital Transformation Process

The concept of burnout, first introduced by Freudenberger (1974) and later systematized by Maslach and Jackson (1981, 1986), has been widely examined across professions such as corporate employees, social workers, lawyers, and healthcare professionals (Pines & Maslach, 1978). Freudenberger, who was the first to label burnout an “occupational hazard,” described it as the depletion of an individual’s internal resources due to unmet expectations, characterized by a loss of energy and strength or by feelings of failure and exhaustion. Building on this foundation, Maslach and Jackson (1986) developed the Maslach Burnout Inventory to enable more structured research, categorizing burnout into three dimensions: depersonalization, emotional exhaustion, and reduced personal accomplishment (Maslach & Jackson, 1978).

Pines and Aronson (1988) defined burnout as a state of physical, emotional, and mental exhaustion resulting from prolonged exposure to emotionally demanding situations. This condition can also be understood as a reaction in which excessive dissatisfaction or stress causes the individual to lose interest in their work. According to Maslach et al. (2014), burnout arises from overcommitment and negatively affects professional life. These authors argue that burnout should not be considered a classical fatigue syndrome but rather a serious psychological disorder that may even result in leaving the profession, reflecting a profound attitudinal shift. Taking a broader perspective, Cardinell (1981) characterized burnout as a severe psychological condition, even equating it to a “midlife crisis.” The literature further suggests that individuals aged between 30 and 35 are under intense pressure due to career expectations, status aspirations, and social challenges, all of which may act as triggers for burnout (Hart, 2020).

Burnout has been defined in various ways in the literature, with no universally accepted definition. This lack of consensus complicates efforts to fully understand its underlying dynamics (Brackett et al., 2010). Burnout is most commonly observed in idealistic individuals working for extended periods in helping professions and emotionally demanding environments. It typically develops insidiously and is strongly associated with long-term occupational stress (Brackett et al., 2010; Cole et al., 2012; Maslach & Jackson, 1981). If its symptoms are ignored, the condition can escalate to an unmanageable level. Early detection and timely preventive action are therefore essential (Brackett et al., 2010). Recent research further highlights that burnout is not only a risk in helping professions but also significantly affects healthcare workers’ mental health worldwide, with chronic exhaustion, cynicism, and reduced professional efficacy consistently reported as outcomes (Icekson et al., 2024).

Burnout syndrome is more frequently observed in individuals who have high expectations of themselves, life, and others (Peterson et al., 2008); care deeply about people and strive to meet their needs (McCornel, 2003); value others (Piko, 2006); take pride in doing their work well (Wissell et al., 2022); set ambitious goals and ideals in life (Qizi & Oglu, 2025); assign meaning to their work beyond financial gain (Petrova & Murzenko, 2018); and tend to blame themselves or engage in negative self-evaluation during times of adversity (Maslach et al., 2014).

Healthcare professionals are among the occupational groups most at risk of burnout (Eriş, 2022; Erdoğan & Üler, 2022; Kılıçarslan & Güçlü, 2019). Contributing factors include excessive stress, the emotional demands of caregiving, inadequate healthcare infrastructure, limited autonomy, low wages, role ambiguity, restricted career advancement, poor professional image, and lack of self-confidence (H. J. Lee, 2018). A scoping review by Nagle et al. (2024) highlights that workplace environment factors—such as role ambiguity, lack of control, and insufficient support—consistently emerge as strong predictors of healthcare worker burnout across diverse settings. Recent findings further suggest that job autonomy can moderate the effect of job demands on burnout, particularly under conditions of high role ambiguity (Reyes-Luján et al., 2025).

Extensive research indicates a direct relationship between burnout and job demands (H. J. Lee, 2018). Physical, emotional, and especially cognitive demands, combined with heavy workloads, create psychological strain and contribute to disengagement from work (Maslach & Jackson, 1981). The digital transformation process further amplifies job demands and may act as a trigger for burnout. In a study of intensive care staff, Van Mol et al. (2015) identified high levels of compassion fatigue and burnout, along with prevalent traumatic stress and a lack of emotional resources. Burnout levels also vary by service group, often peaking among employees with 5–10 years of professional experience (Arnone et al., 2019). Building on this evidence, Zhang et al. (2025) demonstrated that digitalization-related role overload significantly intensifies the effect of job demands on burnout, particularly when job resources are low.

Burnout is one of the most common problems encountered by healthcare professionals (Reid et al., 2010). Rapid changes in today’s healthcare systems have further increased its likelihood within this group. For example, substantial reforms implemented by the Council of Higher Education have raised uncertainty among physicians working as faculty members in medical schools, thereby increasing their risk of burnout (Petrova & Murzenko, 2018). Burnout has become a growing concern among physicians and medical students, typically characterized by emotional exhaustion and reduced professional efficiency. Risk factors include age, work experience, and working conditions, with higher prevalence observed particularly among outpatient clinic staff, teachers, and nurses (Birks et al., 2009; Brackett et al., 2010; Qizi & Oglu, 2025). Because burnout is often associated with social stigma, professionals may be reluctant to seek help (Morse et al., 2012). The strain placed on physicians’ clinical judgment—due to the need to integrate both knowledge and experience—has also been cited as a key contributor to burnout (Khamisa et al., 2015). Studies show that specialist doctors report higher levels of burnout than primary care physicians (Bawack et al., 2021; Dyrbye et al., 2017; Portoghese et al., 2014). In terms of management approaches, non-pharmacological therapies such as hypnotherapy and physical exercise are increasingly used alongside pharmacological treatments (Khamisa et al., 2015).

Healthcare professionals remain at high risk of burnout due to constant exposure to infectious diseases, high expectations from both work and social environments, heavy workloads, and typically insufficient staffing levels (Eriş, 2022; Koutsioumpa, 2023; Pines & Maslach, 1978; Profit et al., 2021). When these challenges are compounded by the demands of digital transformation, the likelihood of burnout in the healthcare sector increases further. Digital transformation in the workplace significantly affects employee well-being and burnout levels. Studies have shown that increasing digital workload and the constant requirement to stay online elevate stress, anxiety, and depression among employees (Agarwal et al., 2010; Alvaro et al., 2022). In their study of German physicians, Müller and Kraus (2024) found that stress-inducing factors such as time pressure and workflow complexity intensified after digital transformation, though stress levels decreased once physicians adapted to digital tools. Similarly, Abdi et al. (2025) reported that although digitally trained Somali healthcare workers experienced increased workloads, their stress levels decreased as they adapted. Doe and Smith (2025) concluded that digitalization may lead to role overload, exacerbating emotional fatigue and burnout. A large-scale study involving 66,000 healthcare workers also reported that the use of Electronic Health Record (EHR) systems resulted in a 40.4% increase in burnout rates (Kim et al., 2024).

### 2.3. Bibliometric Analysis of Concepts

In the healthcare sector, the management of stress and emotions directly affects the quality of service delivery. Examining emotional intelligence and burnout together is therefore of particular importance. This section of the study presents both the results from the Web of Science (WoS) database and the bibliometric analysis conducted using VOSviewer (version 1.6.20). An initial search was planned to include all key concepts—burnout, emotional intelligence, and digital transformation—in the WoS database. However, when the keywords were combined using the “AND” operator across the “topic,” “title,” and “all fields” filters, no studies were found addressing all three concepts simultaneously. Consequently, only the concepts of “burnout” and “emotional intelligence” were included in the search, and the distribution of academic studies addressing both concepts was analyzed by subject area. Designed as a component of the theoretical framework, this analysis provides a systematic overview of how the two concepts are positioned within the literature. The scope of the analysis comprises 540 studies retrieved from the WoS database using the “all fields” filter on 2 February 2025. The search was limited to publications indexed in the SSCI (Social Sciences Citation Index).

#### 2.3.1. Distribution of Studies by WoS Categories

**Figure 1:**
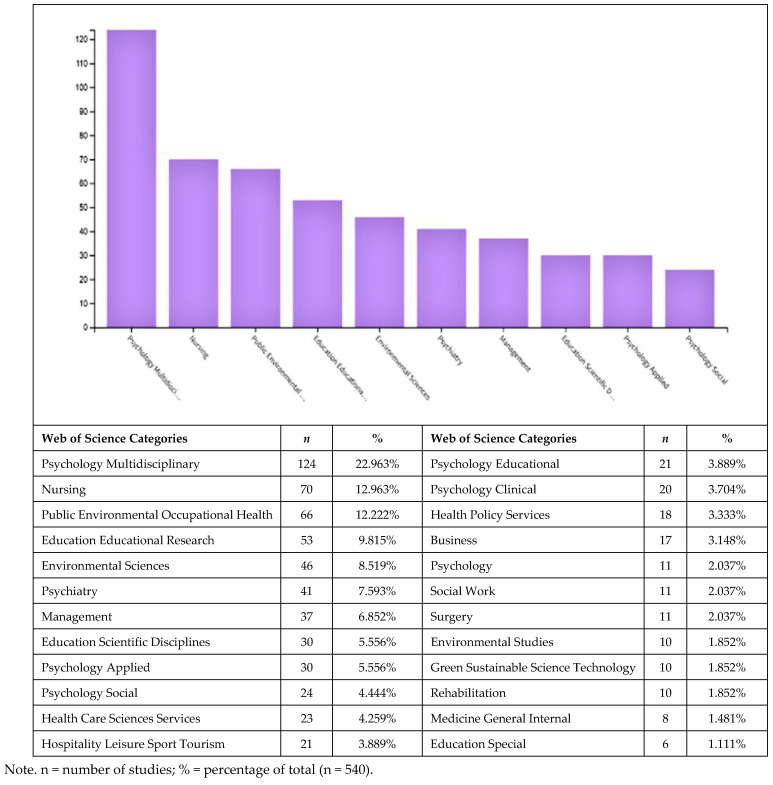
Distribution of the selected studies by Web of Science (WoS) subject categories (n = 540).

The distribution of the selected studies by WoS subject categories is presented in Figure 1. The highest proportion of publications (22.96%) falls under Psychology, Multidisciplinary, followed by Nursing (12.96%), Public, Environmental and Occupational Health (12.22%), and Education, Educational Research (9.81%). Notably, a substantial share of the studies is situated within the field of health.

**Figure 2:**
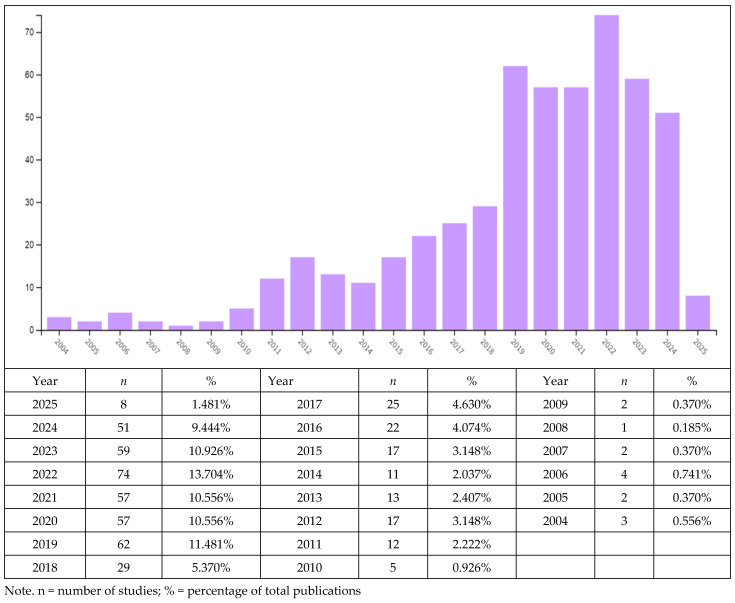
Annual distribution of selected studies on emotional intelligence and burnout by publication year (2004–2025, n = 540).

The distribution of studies by year is shown in Figure 2. The chart reveals a marked increase in publications addressing emotional intelligence and burnout together, particularly over the past five years. The highest number of studies appeared in 2022, with a total of 74 articles (13.70%), followed by 62 articles in 2019 (11.48%), 59 articles in 2023 (10.92%), and 51 articles in 2024 (9.44%). These findings indicate a steadily growing academic interest in jointly studying these two concepts. Notably, their presence in the literature expanded rapidly after the onset of the COVID-19 pandemic.

By contrast, the number of publications before 2015 remained quite limited, with studies mostly confined to individual-level investigations within the field of psychology. This pattern suggests that the combined examination of emotional intelligence and burnout is a relatively recent development that has gained prominence only in the past decade.

#### 2.3.2. Types of Studies

The types of studies are presented in Table 1. As shown in the table, 89.63% (n = 484) of the 540 publications are research articles, while 7.40% (n = 40) are review articles. This pattern indicates that the topic has predominantly been examined through empirical research supported by quantitative data. Other publication types, such as conference abstracts (n = 9), early access articles (n = 7), and editorial materials (n = 6), occur at relatively low rates. These findings suggest that the concepts have been studied systematically and in an evolving manner, with the literature largely shaped by quantitatively grounded articles.

#### 2.3.3. Author Profiles of the Studies

Table 2 presents the author profiles. As shown in the table, the most prolific author is Extremera, Natalio, who has contributed significantly to the literature with 17 publications (3.1%). He is followed by Pérez-Fuentes, M. Carmen with 12 publications (2.2%), Molero Jurado, María del Mar with 11 publications (2.0%), and both Linares, J.J.G. and Mérida-López with 10 publications each (1.8%). Additionally, authors such as Mikolajczak, M., Obeid, S., and Lee, Y.H.—each with seven publications—have also made noteworthy contributions to the literature, particularly within the Asian and European contexts.

**Figure 3:**
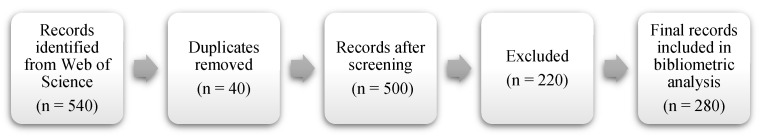
PRISMA-Like Flow Diagram of Bibliometric Study Selection.

Initially, a total of 540 records were identified from the Web of Science database. After removing 40 duplicate records, 500 records remained for screening. Following the screening process, 220 records were excluded, leaving 280 records that met the inclusion criteria and were therefore included in the bibliometric analysis. The search, screening, and inclusion process is illustrated in Figure 3 as a PRISMA-like flow diagram.

#### 2.3.4. VOSviewer Co-Authorship Analysis

**Figure 4:**
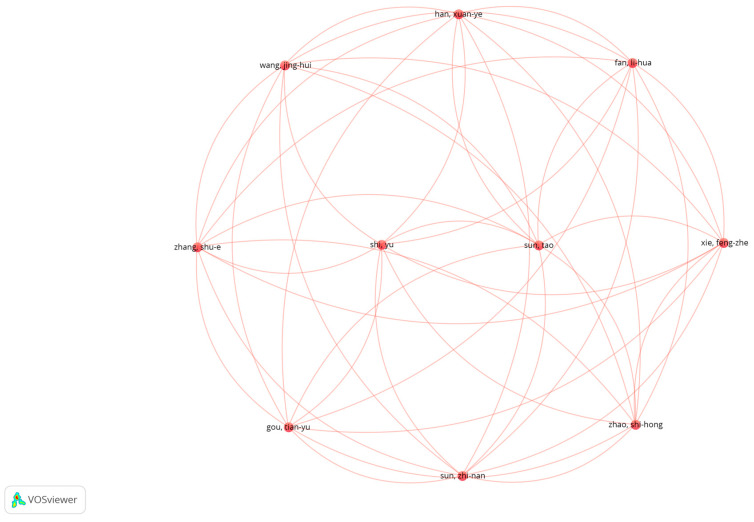
Co-authorship Network Showing Collaboration Among Authors.

This analysis examines the collaboration networks of authors working in the fields of emotional intelligence and burnout. Using VOSviewer, a network map was generated based on the criteria of at least one publication and one citation, resulting in the inclusion of 168 authors. The co-authorship network among these authors is visualized in Figure 4. According to the analysis, 10 clusters, 310 links, and a total link strength of 314 were identified. Notable author pairs with strong co-authorship ties include Beaumont & Elaine, Carson & Jerome, Durkin & Mark, Martin & Caroline J., Hollins & Extremera, and Natolio & Lee Junghoon. Each of these pairs has collaborated on at least two articles, jointly contributing to the literature.

Authors positioned closer to the center of the network tend to have collaborated with a larger number of colleagues, thereby expanding the overall collaboration network.

#### 2.3.5. VOSviewer Citation Analysis of Authors

**Figure 5:**
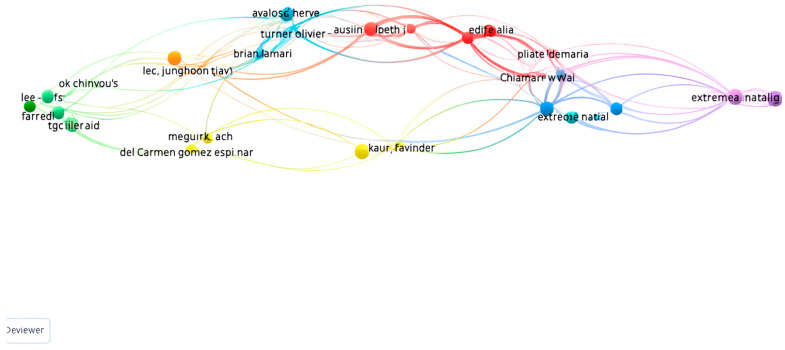
Citation Network of Authors.

A citation analysis was conducted to identify the most influential academic contributions related to the studied concepts. Based on the criteria of at least one publication and one citation, the resulting network comprised 7 clusters, 79 items, 351 links, and a total link strength of 359. The highest citation count was recorded by the author pairs Aguinis & Herman and Bernerth & Jeremy, each with 1167 citations. They were followed by Bakker & Arnold and De Vries & Juriena, each with 440 citations, and by Lee, Junghoon with 362 citations. Other prominent pairs include Bakker & Jan, Benoit & Dominique, Kompanje & Erwin, Nijkamp & Marjan, and Van Mol & Margo, each with 360 citations. The citation network of these authors is illustrated in Figure 5.

#### 2.3.6. Keyword Analysis (Co-Occurrence of All Keywords)

This analysis explores keyword-level patterns in studies involving the relevant concepts. A total of 18 clusters, 128 items, 490 links, and a total link strength of 537 were identified. The most frequently used keywords were “emotional intelligence” (20 occurrences) and “burnout” (18 occurrences). These two concepts appeared in separate clusters but were connected to numerous other terms. The map shows that both concepts are positioned at the center and form the backbone of the network structure.

**Figure 6:**
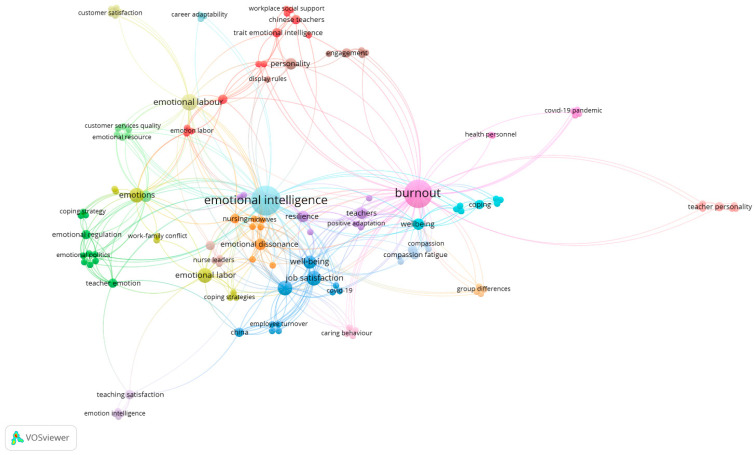
Co-occurrence Map of the Most Frequently Used Keywords.

Keywords such as empathy, coping, well-being, work engagement, job satisfaction, and emotional labor serve as bridges between emotional intelligence and burnout. Among the 18 identified clusters, four stood out for their dense connectivity: (1) the context of healthcare workers, (2) education-focused studies, (3) psychological resilience, and (4) leadership–empathy interactions. The analysis reveals that emotional intelligence and burnout are not only linked at the individual level but also embedded within complex, profession-related relationship networks. The keyword co-occurrence network is presented in Figure 6.

#### 2.3.7. VOSviewer Country Citation Analysis

**Figure 7:**
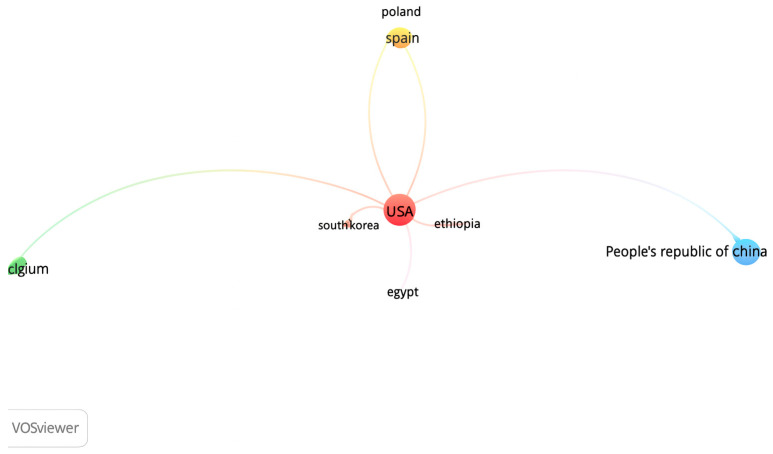
Country Citation Network.

This analysis examined the citation relationships based on the countries of the authors’ affiliations, using the criteria of at least one publication and one citation. A total of 128 countries were identified, grouped into 18 clusters, with 490 links and a total link strength of 537. The countries with the highest number of publications were the United States (15), followed by the United Kingdom (8), China (6), and both Spain and Germany (5 each). In terms of citations received, the United States ranked first with 3236 citations, followed by the Netherlands (1067), Belgium (814), the United Kingdom (696), Spain (804), China (740), Scotland (642), and Poland (331). The resulting network structure shows that citations are predominantly concentrated around publications originating from Western Europe and North America. The citation network among countries is illustrated in Figure 7.

#### 2.3.8. VOSviewer Bibliographic Coupling Analysis of Documents

Bibliographic coupling occurs when two different publications cite the same reference. In this analysis, 50 publications with at least one citation were examined. The results revealed 4 clusters, 599 links, and a total link strength of 1549. The strongest couplings were observed for the following: J. J. Lee and Ok (2012), with 206 citations and a link strength of 223; Lee, with 156 citations and a link strength of 1545; Mérida-López and Extremera (2017), with 159 citations and a link strength of 143; and Ju et al. (2015), with 112 citations and a link strength of 135.

**Figure 8:**
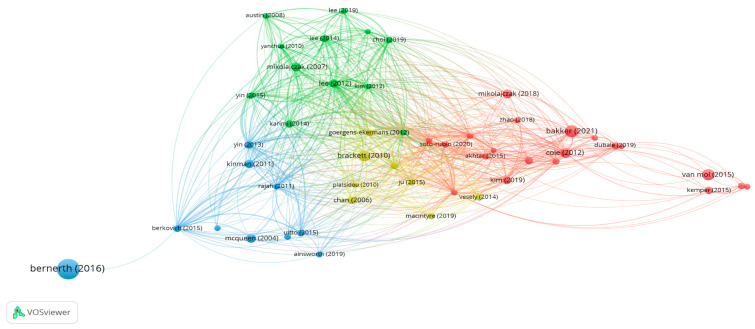
Bibliographic Coupling Network of Documents.

These findings indicate that studies on emotional intelligence and burnout are grounded in shared references and that certain sources occupy a central position in the literature. The bibliographic coupling network of the documents is presented in Figure 8.

#### 2.3.9. VOSviewer Bibliographic Coupling Analysis of Authors

This analysis examined the extent to which authors cite common sources, using the criteria of at least one publication and one citation. A total of 168 publications were included, revealing 14 clusters, 6998 links, and a total link strength of 40,915. The strongest bibliographic couplings were observed between Lee & Junghoon, with 362 citations and a link strength of 1292, and Alem & Atalay, with 135 citations and a link strength of 1188. Other notable pairings include Chermali & Zeina, Denninger & John, Dubale & Benyam, Fricchione & Gregory, Friedman & Lauren, Gelaye & Bizu, and Mehta & Darshan.

**Figure 9:**
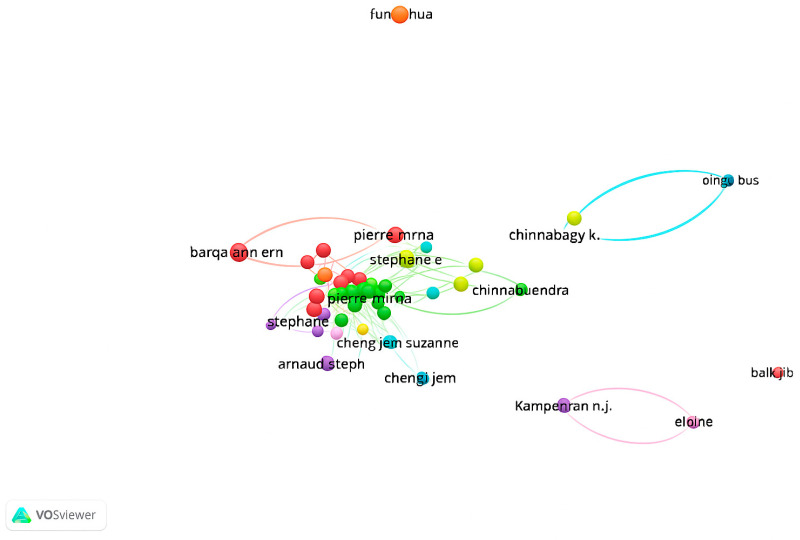
Bibliographic Coupling Network of Authors.

This network shows that authors draw upon similar bodies of literature and that specific pairings are particularly prominent within their respective clusters. The bibliographic coupling map of authors is presented in Figure 9.

#### 2.3.10. VOSviewer Co-Citation Analysis of Authors

Authors who are cited together within the same study provide valuable insights into the theoretical proximity of the literature. Using a minimum threshold of ten citations, the analysis identified 25 author units forming 3 clusters, with 276 links and a total link strength of 3189.

**Figure 10:**
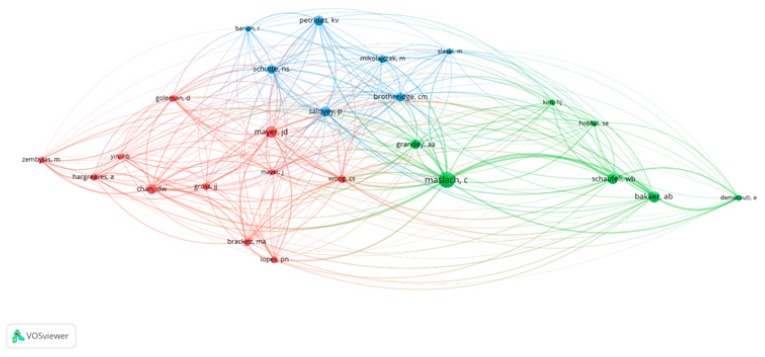
Co-Citation Network Among Frequently Cited Authors.

The resulting structure shows that certain authors are frequently cited together, underscoring their foundational role in shaping the field. The co-citation network among authors is visualized in Figure 10.

The most frequently cited authors were Maslach, with 65 citations and a total link strength of 765; Mayer, with 34 citations and a link strength of 437; Bakker, with 33 citations and a link strength of 408; and Schaufeli, with 25 citations and a link strength of 382.

All analyses conducted in this study reveal the multidimensional positioning of the key concepts within the literature. The findings allow for the observation of both individual and institutional scholarly production and interaction, clearly identifying trends, collaboration patterns, and focal research areas. Prominent figures such as Mayer, Schaufeli, and Maslach emerged as key contributors, with most studies concentrated in the United States, the United Kingdom, and the Netherlands.

From a conceptual standpoint, in addition to the primary keywords, frequently co-occurring terms included emotional labor, job satisfaction, and well-being. Overall, the results of these analyses provide not only a structural map of the existing literature but also a strong foundation for guiding future research.

### 2.4. Theoretical Framework Within the Context of the Job Demands-Resources (JD-R) Model

The Job Demands–Resources (JD-R) model is one of the most widely used theoretical frameworks for explaining employees’ psychosocial conditions (Bakker et al., 2004; Demerouti et al., 2001). It is grounded in the balance between the demands employees face and the resources they possess (Bakker & de Vries, 2021). Job demands refer to aspects of work that require sustained psychological effort, whereas the psychological and physical elements that help individuals cope with these demands are referred to as job resources (Bakker et al., 2014).

According to the model, demands such as technological changes, time pressure, and workload can lead to psychological and physical costs (Bakker et al., 2014; Demerouti et al., 2001), whereas job resources—such as social support, emotional intelligence, and competence (Schaufeli, 2017; Van Wingerden et al., 2017)—can mitigate these costs (Cheng et al., 2023), enhance job satisfaction, and increase motivation (Bakker & Demerouti, 2017).

The JD-R model has been frequently applied in the healthcare sector, particularly in relation to individual resources such as emotional labor (Bakker & Demerouti, 2017; Choi et al., 2019), stress management (Hart, 2020; Portoghese et al., 2014), and empathy (Bar-On, 1997; Sahney et al., 2024), as well as to demands like uncertainty and workload (Alvaro et al., 2022).

Within the JD-R framework of this study, the expectations for technological adaptation brought about by digital transformation are conceptualized as a job demand, while emotional intelligence is regarded as an individual resource. It is assumed that an imbalance between job demands and personal resources may increase burnout, and in this context, emotional intelligence is evaluated as a potential buffe.

**Figure 11:**
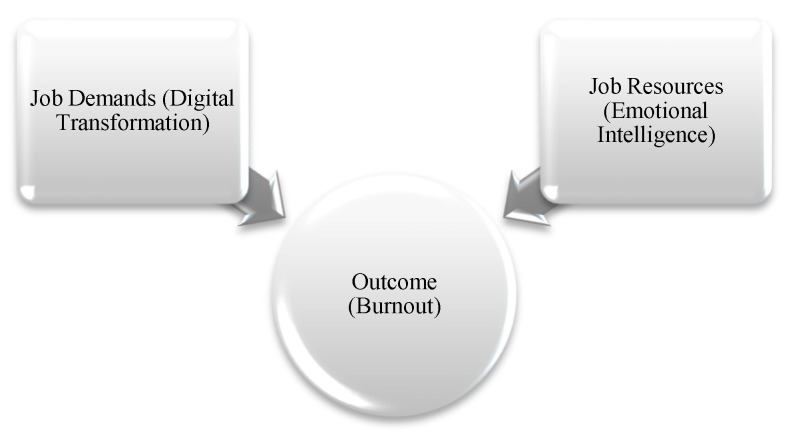
Visual Representation of the JD-R Model Adapted to the Current Study. Source: Adapted from Bakker and Demerouti (2017).

Figure 11 presents a visual adaptation of the JD-R Model aligned with the scope of this study.

The JD-R model explains employee behavior in the context of job demands and resources. Within the framework of this study, technological transformation and its requirements are conceptualized as job demands, while emotional intelligence is treated as a job resource. In this research, the JD-R model is not used as a measurable construct but rather as a theoretical framework that defines the conceptual foundation of the study. The job-demand dimension of the model is associated with the digital adaptation process, while the resource component is linked to emotional intelligence.

In other words, the outcome variable—burnout—is evaluated in relation to the workload caused by digitalization and the protective role of individual resources. Although no direct, measurable questions regarding the challenges of digital transformation were posed to participants, care was taken to select healthcare institutions that had recently undergone a digital transition.

## 3. Materials and Methods

This section elaborates the overall research approach, data collection tools, implementation process, analysis methods, and sample characteristics. This descriptive cross-sectional research was conducted among healthcare staff working at four public hospitals in Turkey that had progressed from HIMSS Stage 6 to 7. A total of 590 participants were selected using a convenience sampling method. Data were collected via a structured questionnaire consisting of three parts: (i) a “Demographic Information Form” to assess participants’ gender, age, marital status, years of service, and monthly income; (ii) the “Maslach Burnout Inventory”; and (iii) the “Schutte Emotional Intelligence Scale.” Statistical analyses were performed to examine the relationships among the variables.

This study adopted a mixed-methods design. The qualitative component was represented by a bibliometric analysis of existing studies, and the quantitative component consisted of a structured questionnaire administered to healthcare professionals. Because no interviews were conducted, open-ended questions were not included. The key constructs of the model were operationalized through standardized scales (Maslach Burnout Inventory and Schutte Emotional Intelligence Scale), whose sub-dimensions and item numbers are presented in Table 3 and Table 4. This design was chosen to provide a more comprehensive understanding of the relationship between emotional intelligence and burnout during digital transformation, as quantitative data alone may not capture the contextual and experiential aspects of the phenomenon.

In this paper, the term “mixed-methods” refers specifically to the combination of a bibliometric analysis and a quantitative survey. No interviews, open-ended questions, or other qualitative data collection techniques were employed. Thus, the study should be understood as primarily quantitative, supplemented by a bibliometric mapping of the literature rather than a full qualitative component.

### 3.1. Type and Purpose of the Research

This research was conducted to assess the emotional intelligence and burnout levels of healthcare professionals working in four public hospitals in Turkey that had recently transitioned from HIMSS Stage 6 to Stage 7 a benchmark of significant digital health transformation. The evaluation was carried out particularly in the context of demographic characteristics and aimed to explore the relationship between the two constructs.

The study was structured around two main objectives. In the first phase, a bibliometric analysis of the literature on emotional intelligence and burnout was performed to map conceptual clusters, academic collaborations, and citation patterns in the field. Although each of the three key concepts-digital transformation, emotional intelligence, and burnout-has been widely studied independently, the combination of emotional intelligence and burnout appears together in only a limited number of studies. No studies incorporating all three concepts simultaneously were found in the Web of Science (WoS) database. This finding highlights a significant gap in the literature and underscores the importance of the second phase of the research.

In the second phase, the relationship between emotional intelligence and burnout among healthcare professionals actively experiencing digital transformation was analyzed. The study employed a correlational and descriptive research design. In this context, the relationships between the sub-dimensions of burnout (personal accomplishment, depersonalization, and emotional exhaustion) and the sub-dimensions of emotional intelligence (mood regulation/optimism, use of emotions, and appraisal of emotions) were examined in relation to participants’ demographic characteristics.

### 3.2. Research Model and Hypotheses

The literature includes several studies demonstrating a negative relationship between emotional intelligence (EI) and burnout. For example, Williams-York et al. (2024) found a strong negative correlation between EI and burnout among healthcare students. Similarly, Y. Cao et al. (2022) emphasized the mitigating role of EI in burnout among healthcare professionals, particularly highlighting a significant interaction between EI and the sub-dimensions of depersonalization and emotional exhaustion. In another study, Szczygiel and Mikolajczak (2018) underscored the buffering effect of EI in helping nurses cope with negative emotions.

**Figure 12:**
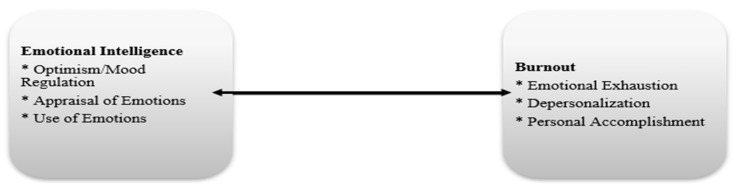
Research Model.

Guided by these findings, the present study also investigates the relationship between EI and burnout, offering an original contribution by focusing on healthcare personnel working in Turkish public hospitals that have recently undergone digital health transformation. These examples from the literature provided the basis for shaping the research model. Accordingly, the theoretical model of the study is presented in Figure 12.

Based on the model, the hypothesis of the study is as follows:

**H1:**  *There is a statistically significant relationship between emotional intelligence and burnout levels among healthcare professionals.*

### 3.3. Data Collection Tools

In this study, a Socio-Demographic Information Form consisting of five questions was developed by the researchers to collect basic demographic data from the participants, including gender, age, marital status, years of work experience, and monthly income. To assess emotional intelligence and burnout levels, two separate scales-each proven to be valid and reliable in the literature-were employed. Burnout levels were measured using the Maslach Burnout Inventory (MBI), originally developed by Maslach and Jackson (1981) and adapted into Turkish by Çam (1992). This scale consists of 22 items and evaluates three subdimensions: emotional exhaustion, depersonalization, and personal accomplishment. Items are scored on a 5-point scale ranging from 0 to 4 (0 = never, 1 = rarely, 2 = sometimes, 3 = often, 4 = always).

Emotional intelligence levels were measured using the Schutte Emotional Intelligence Scale, developed by Schutte et al. (1998) and adapted into Turkish by Tatar et al. (2016). This 41-item scale assesses participants’ abilities to perceive, regulate, and use emotions. It is structured with a 5-point Likert scale (1 = strongly disagree, 2 = disagree, 3 = neutral, 4 = agree, 5 = strongly agree) and comprises three main subdimensions: optimism/mood regulation, use of emotions, and appraisal of emotions. Table 3 and Table 4 present the subdimensions of the scales used in the study, along with the corresponding questionnaire items.

In this study, burnout was measured using the Maslach Burnout Inventory (MBI), which comprises three subdimensions: Emotional Exhaustion, Depersonalization, and Personal Accomplishment. Higher scores on Emotional Exhaustion and Depersonalization indicate higher burnout, whereas higher scores on Personal Accomplishment indicate lower burnout. Emotional intelligence was assessed using the Schutte Emotional Intelligence Scale, which includes the subdimensions of Optimism, Evaluation of Emotions, and Use of Emotions. All items were scored on a Likert scale, and negatively worded items were reverse-coded prior to analysis.

Emotional intelligence was specifically measured using the Turkish version of the Schutte Emotional Intelligence Scale adapted by Tatar et al. (2016), which consists of 36 items. The three subdimensions used in this study—Use of Emotions, Appraisal of Emotions, and Mood Regulation/Optimism—were determined based on the factor structure reported in this Turkish adaptation.

### 3.4. Data Collection and Analysis

The data for this study were collected using questionnaire forms hand-delivered in sealed envelopes to healthcare professionals working in all departments by the researchers. Approximately ten days after distribution, the completed questionnaires were collected and the data analysis process was initiated. The collected data were entered into SPSS 23.0 and analyzed using this program.

Population and Sample of the Study

This study employed a descriptive and correlational survey design focusing on healthcare professionals working in four public hospitals that had transitioned from HIMSS Stage 6 to Stage 7. The target sample size was calculated as 600 individuals at a 95% confidence level with a 4% margin of error, based on the sample size table provided by Yazıcıoğlu and Erdoğan (2004). The final sample consisted of 590 healthcare professionals who voluntarily participated in the study, corresponding to a response rate of approximately 98% of the targeted number. Participants were selected using a convenience sampling method.

Reliability analyses indicated that Cronbach’s alpha values for the subscales ranged from 0.78 to 0.91, demonstrating good internal consistency. Confirmatory factor analysis supported the validity of the measurement model with acceptable fit indices (χ^2^/df = 2.34, RMSEA = 0.058, CFI = 0.95, TLI = 0.94). Reverse-coded items were recoded prior to analysis, and subscale scores were computed by averaging the relevant items. All statistical assumptions (normality, homogeneity of variance, and multicollinearity) were checked before analysis, and effect sizes (η^2^, Cohen’s d, and R^2^) were reported alongside the results. According to Cohen’s (1988) guidelines, the correlation coefficient (r = −0.317) represents a medium-strength relationship. The minimum sample sizes required at different error and confidence levels for a population larger than 500,000 are shown in Table 5.

During the research process, written authorization was obtained from the chief physicians of the participating healthcare institutions, and informed consent was secured from all participants on a voluntary basis. Throughout the study, the confidentiality of personal data was strictly maintained, and ethical principles were meticulously observed. Because this investigation was non-interventional and descriptive in nature, approval from an ethics committee was not required.

### 3.5. Data Analysis

The collected data were transferred to SPSS 23.0 for statistical analysis. To determine whether emotional intelligence and burnout levels differed according to participants’ demographic characteristics, independent-samples *t*-tests and one-way analysis of variance (ANOVA) were first conducted. Next, to assess the overall relationship between emotional intelligence and burnout, Pearson correlation analysis was performed, allowing the direction and significance of the association between the key variables to be evaluated. Finally, multiple regression analyses were conducted to examine the extent to which the subdimensions of emotional intelligence predicted the subdimensions of burnout, thereby providing a detailed evaluation of both relationships and predictive effects.

## 4. Results

This section presents the descriptive statistics, difference analyses, general correlation results, relationships among subdimensions, and regression analysis outcomes.

### 4.1. Descriptive Statistics

A majority of the study participants were women, accounting for 67.2% of the sample, while men comprised 32.8%. In terms of age distribution, participants aged 20–39 were similarly represented across groups. Nearly half of the sample (47.3%) reported 10 or more years of professional experience, and 56.8% identified as single. Regarding income levels, 28.3% of the participants reported earning a monthly salary between 50.001 and 75.000 TRY.

### 4.2. Difference Analyses

The relationships between socio-demographic variables (e.g., gender, age, work experience, marital status) and the subdimensions of the Maslach Burnout Inventory-Emotional Exhaustion, Insensitivity, and Personal Success-were analyzed using independent-samples *t*-tests for two-group comparisons and one-way analysis of variance (ANOVA) for multi-group comparisons. A significance level of *p* < 0.05 was adopted. The results are presented in Table 6.

When examined by subdimensions, no statistically significant differences were found between female and male healthcare professionals in Maslach Burnout Inventory (MBI) scores. However, analysis of the age variable revealed statistically significant differences in MBI scores (all *p*-values < 0.05). The 20–29 age group had the highest scores across all subscales-Emotional Exhaustion (EE), Depersonalization (DP), and Personal Accomplishment (PA)-while the 50+ age group had the lowest scores, indicating that burnout levels decrease with increasing age.

Overall, healthcare professionals aged 50 and older demonstrated lower levels of burnout. Significant differences were also observed in MBI scores by years of professional experience. The highest levels of burnout were found among those with 0–4 years of experience, while the lowest levels were observed among those with more than 10 years, suggesting that burnout decreases as professional tenure increases.

No statistically significant difference was found in burnout scores based on income levels. The relationship between socio-demographic characteristics and the subdimensions of the Emotional Intelligence Scale is presented in Table 7.

When examining the gender variable, statistically significant differences were observed in the subdimensions of Optimism/Mood Regulation (OMR) and Use of Emotions (UE) (*p* = 0.001). In both dimensions, female participants scored higher than males, suggesting that women possess higher levels of emotional intelligence in terms of managing emotions and maintaining a positive emotional state. No significant difference was found in the Appraisal of Emotions (AE) dimension (*p* = 0.433).

Regarding age, a significant difference was found only in the OMR dimension (*p* = 0.013). The highest scores were observed in the 50+ age group, while the lowest scores were recorded in the 20–29 age group, suggesting that emotional stability and the ability to maintain a positive mood may strengthen with age.

In terms of years of professional experience, a statistically significant difference was also found exclusively in the OMR subdimension (*p* = 0.012). Participants with 0–4 years of experience had the lowest scores, while those with more than 10 years had the highest scores, indicating that professional experience contributes to emotional flexibility.

Based on marital status, significant differences were found in the AE (*p* = 0.010) and UE (*p* = 0.003) dimensions. Single individuals scored higher in both dimensions, which may suggest that their emotional intelligence is enhanced through more dynamic or varied social interactions.

Regarding income, a statistically significant difference was found only in the UE dimension (*p* = 0.004), indicating that an individual’s economic situation may influence their capacity to effectively utilize emotions.

### 4.3. General Correlation Results

This section presents the results of the Pearson correlation analysis conducted to determine the overall relationship between emotional intelligence and burnout levels. The Pearson correlation results regarding the relationship between emotional intelligence and burnout are shown in Table 8.

According to the findings, a significant negative correlation was observed between emotional intelligence and burnout levels (r = −0.317; *p* < 0.05). This result indicates that as emotional intelligence increases, burnout decreases. Therefore, Hypothesis H is supported. This finding is consistent with previous studies in the literature, such as those conducted by Williams-York et al. (2024) and Y. Cao et al. (2022).

### 4.4. Relationships Among Subdimensions

Based on the significant correlations observed, the predictive effects of the subdimensions of emotional intelligence on the subdimensions of burnout were tested using regression analysis. The relationships between the subdimensions of emotional intelligence and those of the Maslach Burnout Inventory were examined, and the results of the Pearson correlation analysis are presented.

This analysis reveals both the direction and the significance level of the relationships between subdimensions. The results regarding the relationships between emotional intelligence and burnout subdimensions are presented in Table 9.

According to the table, the Optimism/Mood Regulation subdimension showed significant negative correlations with Emotional Exhaustion (r = −0.150), Depersonalization (r = −0.278), and Personal Accomplishment (r = −0.317), indicating that as individuals’ levels of optimism increase, their levels of burnout tend to decrease.

Similarly, the Appraisal of Emotions subdimension was positively correlated with Emotional Exhaustion (r = 0.238) and Depersonalization (r = 0.224), and negatively correlated with Personal Accomplishment (r = −0.416), all of which were statistically significant.

The Use of Emotions subdimension, however, did not show any significant relationship with any of the burnout subdimensions. This suggests that certain dimensions of emotional intelligence may exert a stronger influence on burnout levels than others.

Within the framework of the Job Demands–Resources (JD-R) Model, these findings support theoretical expectations. While the demands created by digital transformation—such as increased workload and pressure to adapt to technology—elevate the risk of burnout among healthcare professionals, emotional intelligence functions as a personal resource that buffers these adverse effects. The significant negative correlation between emotional intelligence and burnout (r = −0.317; *p* < 0.05) confirms the model’s proposition that resources play a mitigating role in reducing burnout.

Furthermore, the subdimension analyses show that Optimism/Mood Regulation and Appraisal of Emotions have particularly strong effects on Depersonalization and Personal Accomplishment, suggesting that some personal resources may offer stronger protective effects against specific job demands.

These results underscore the importance of emotional intelligence training and mentoring programs, especially in the context of digital transformation in healthcare settings.

## 5. Discussion

Emotional intelligence (EI) is considered a protective factor, particularly for healthcare professionals working in high-stress environments. Numerous studies have shown that higher levels of EI are associated with lower levels of burnout (Arnone et al., 2019; Vlachou et al., 2016). Weng et al. (2011) found that EI positively affects job satisfaction, patient satisfaction, and burnout. Pau and Croucher (2003) reported that EI helped reduce occupational stress among dental students, while Birks et al. (2009) demonstrated that individuals with low stress levels were better able to use EI to cope with challenges. Furthermore, technostress arising during digital transformation processes has been shown to increase burnout, but EI can buffer this effect (Shaban et al., 2025).

The “Use of Emotions” subdimension emerged as a particularly critical component of healthcare professionals’ EI repertoire during digital transformation. Although its association with burnout appeared weaker compared to other subdimensions, several contextual factors may underlie this finding. First, healthcare workers operating in highly regulated hospital environments may consciously or unconsciously underreport their use of emotions, resulting in response bias (Podsakoff et al., 2003) that can obscure actual behavior. Second, occupational factors such as differences in workload between departments, hierarchical structures, intensity of patient interactions, and shift schedules may restrict the functionality and visibility of this dimension (Leiter & Maslach, 2016). Third, institutional influences such as organizational culture, integration of digital workflows, managerial style, and technological support levels constitute key contextual conditions that determine how effectively emotions can be used in workplace processes.

Within the Job Demands–Resources (JD-R) model, the “Use of Emotions” subdimension can be interpreted as a personal resource enabling employees to cope with the pressures and stress created by digital job demands (Bakker & Demerouti, 2017; Demerouti et al., 2001). However, when personal resources are constrained by organizational and occupational conditions, the protective and restorative effect of this dimension against burnout may be weakened. These findings underscore the importance for healthcare institutions undergoing digital transformation to implement targeted training programs, mentorship, simulation-based practices, and organizational support mechanisms to enhance the use of emotions as a resource. In this way, the “Use of Emotions” subdimension can be considered not only an individual skill but also a strategic resource that affects organizational performance and employee well-being.

A hybrid intervention program developed by Makowska-Tłomak et al. (2022) was found effective in reducing stress and burnout related to digital transformation. Similarly, Ertiö et al. (2024) emphasized the importance of digital leadership and employee support in alleviating burnout. These findings highlight the necessity of integrating EI into managerial strategies in the digital age. Demographic factors such as age, marital status, and work environment may also influence burnout levels. However, EI appears to serve as a buffer, enhancing employees’ psychological resilience in the face of these factors (Antoniou & Koronaiou, 2018).

In this study, the selection of hospitals at HIMSS Stages 6–7 was used as an organizational-level indicator of digital transformation. However, HIMSS stages do not directly measure how employees are individually affected by digital transformation. Therefore, while the findings provide strong contextual evidence that digital transformation increases job demands and the risk of burnout, they do not allow direct inferences about the magnitude or direction of this effect at the individual level. As such, the findings should be interpreted by considering digital transformation as an environmental factor and understanding the EI–burnout relationship within this context.

While this approach offers contextual evidence that digital transformation increases job demands, it does not directly measure the magnitude or direction of employees’ individual experiences. Future research should employ standardized scales or multi-source data to capture individual-level digitalization effects more accurately.

## 6. Conclusions

Organizational behavior has long been a field of research with strategic importance for institutions (Erdoğan & Üler, 2022). Burnout is one of the most extensively studied topics within psychology and is typically defined as a chronic stress syndrome (Bakker et al., 2014; Maslach et al., 2001). Mikolajczak et al. (2007) emphasized the protective role of emotional intelligence (EI) in mitigating burnout, while McQueen (2004) demonstrated the determinant role of EI in nurses’ ability to perform their duties effectively.

In the healthcare sector, high workloads and a strong sense of responsibility often lead to both physical and mental fatigue among employees. Symptoms such as sleep disturbances, depressed mood, and social withdrawal manifest as burnout and are further exacerbated by difficulties in achieving work–life balance and perceived lack of career advancement. Among healthcare professionals, burnout tends to be more complex due to the diversity and intensity of occupational responsibilities, directly impacting professional performance.

With the rise of digital transformation, healthcare workers face increasing pressure to continuously adapt to new technologies. This pressure generates stress and uncertainty, which in turn challenge emotional resilience. In this context, the present study goes beyond quantitative findings by incorporating bibliometric analysis to explore conceptual relationships in the literature, thereby adding theoretical depth.

In addition to empirical data, the study evaluated EI and burnout within the framework of academic collaborations and conceptual clustering, offering insights into psychosocial challenges faced by healthcare workers during digital transformation. These findings not only identify critical factors contributing to coping mechanisms but also provide theoretical and methodological guidance for future research.

It has therefore become increasingly important not only to examine the influence of EI on healthcare workers’ professional lives but also to assess its potential to reduce burnout during digital transformation processes. Work-related stress is particularly common in professions involving intense interpersonal interaction. Among healthcare professionals, this often results from the burden of patient care, the demand for emotional support, and high workload—factors that contribute to professional dissatisfaction and eventually burnout (Petrova & Murzenko, 2018).

The bibliometric analysis conducted via Web of Science (WoS) revealed that the concepts of EI and burnout are studied not only in psychology but also across multiple disciplines, including health, social sciences, education, and management. The concentration of studies in fields such as nursing, environmental health, and psychiatry underscores the relevance of focusing on healthcare professionals. The increase in publications after 2020 is believed to be related to heightened stress levels among healthcare workers during the COVID-19 pandemic.

The analysis further showed that most of the prominent authors are affiliated with universities in Spain and work collaboratively in fields such as healthcare, psychology, and education. Co-authorship and citation analyses revealed that the literature is shaped by specific academic clusters, indicating strong research collaboration in the field. The keyword analysis highlighted terms such as “COVID-19 pandemic,” “healthcare professionals,” and “compassion fatigue,” reflecting the strong emphasis on healthcare workers in the literature.

These findings extend previous research by demonstrating that emotional intelligence is a key personal resource that buffers the negative effects of digital transformation on burnout, in line with the JD-R model. Compared with earlier studies focusing solely on emotional intelligence or burnout separately, our results provide integrative evidence within the healthcare context. This underscores the need for organizations to incorporate EI-focused interventions and training programs into digital transformation initiatives. Future research should explore the long-term impact of such interventions and test the proposed relationships across different healthcare systems.

### 6.1. Limitations

This study has several limitations. First, the bibliometric analysis was limited to the Web of Science (WoS) database and the SSCI category, which may have constrained its depth by excluding relevant research from other databases. Moreover, digital transformation was not included as a keyword in the bibliometric analysis and was addressed only within the theoretical framework, which may have limited the conceptual scope and leaves room for further investigation.

Second, the field study was conducted in only four hospitals. The use of sealed-envelope surveys and the short response period may have reduced participant motivation. Furthermore, digital transformation was not quantitatively measured; instead, the HIMSS Stages 6–7 status of the hospitals served only as an organizational-level proxy. Employees’ actual digital workload, technostress, or system usage intensity were not assessed, necessitating that the findings be interpreted as associative rather than causal. Future research would benefit from developing or employing standardized tools to measure the individual-level impact of digital transformation to enhance the accuracy and generalizability of results.

Third, emotional intelligence was measured using the Schutte Emotional Intelligence Scale, a widely used trait-based instrument. Some trait-based frameworks suggest that emotional intelligence is relatively stable and not easily modifiable. Therefore, the results of this study should be interpreted with the understanding that the measured construct reflects more of an individual characteristic rather than a readily changeable skill.

Finally, the study relied on a cross-sectional, self-report design, which may be subject to social desirability bias, common-method variance, and unmeasured confounders (e.g., department type, workload intensity, or managerial style). This context-specific sampling strategy and design limit the ability to draw firm causal conclusions and to generalize the findings to other healthcare systems or cultural settings.

### 6.2. Recommendations

Healthcare institutions should organize training sessions and workshops aimed at enhancing employees’ emotional intelligence. These programs should address essential competencies such as stress management, emotional regulation, empathy, and self-awareness. In particular, resilience-building and awareness-raising initiatives should be implemented during the preparation phase of digital transformation.

At the organizational level, strengthening support systems, expanding access to psychological counseling, and implementing work–life balance policies may help healthcare professionals manage burnout more effectively.

For future studies, it is recommended to:

- incorporate multiple data sources beyond WoS;
- conduct fieldwork in different types of healthcare institutions; and
- examine digital transformation as a direct variable.

Such an approach would lead to more comprehensive, representative, and generalizable findings.

Based on the findings of this study, younger and less experienced healthcare professionals appear to be at higher risk for emotional exhaustion and depersonalization. Therefore, emotional resilience training, mentoring systems, and psychosocial support mechanisms should be developed specifically for professionals with 0–4 years of experience during digital transformation. These preventive strategies could reduce burnout while enhancing employee engagement and a sense of personal accomplishment.

In future research, it would also be beneficial to analyze the effects of digital transformation on burnout and emotional intelligence using bibliometric methods. Furthermore, specific management strategies for technostress should be developed to support employees throughout the digital transformation process.

## Figures and Tables

**Table 1:** Types of publications on emotional intelligence and burnout in the Web of Science (n = 540).

Record Count	*n*	% of 540	Record Count	*n*	% of 540
Article	484	89.630%	Editorial Material	6	1.111%
Review Article	40	7.407%	Proceeding Paper	2	0.370%
Meeting Abstract	9	1.667%	Book Chapters	1	0.185%
Early Access	7	1.296%	Correction	1	0.185%

Note. n = number of publications; % = percentage of total records (n = 540).

**Table 2:** Most prolific authors publishing on emotional intelligence and burnout (n = 540).

Name	*n*	%	Name	*n*	%
Extremera N	17	31.48%	Haddad C	6	11.11%
Pérez-fuentes MD	12	22.22%	Salameh P	6	11.11%
Jurado MDM	11	20.37%	Chambel MJ	5	9.26%
Linares JJG	10	18.52%	Fiorilli C	5	9.26%
Mérida-lópez S	10	18.52%	Fernández-berrocal P	5	9.26%
Rey L	8	14.81%	Liu L	5	9.26%
Hallit S	7	12.96%	Martínez AM	5	9.26%
Lee YH	7	12.96%	Prentice C	5	9.26%
Mikolajczak M	7	12.96%	Roskam I	5	9.26%
Márquez MDS	7	12.96%	Shkoler O	5	9.26%
Obeid S	7	12.96%	Wu H	5	9.26%
Akel M	6	11.11%	Carvalho VS	4	7.41%
Fares K	6	11.11%			

Note. n = number of publications by the author; % = percentage of total records (n = 540).

**Table 3:** Maslach Burnout Inventory (MBI) Sub-Dimensions and Corresponding Survey Items.

Sub-Dimensions	Survey Questions
Emotional Exhaustion (EE)	1, 2, 3, 6, 8, 13, 14, 16, 20
Insensitivity (I)	5, 10, 11, 15, 22
Personal Success (PS)	4, 7, 9, 12, 17, 18, 19, 21

Note. EE = Emotional Exhaustion; I = Insensitivity; PS = Personal Success.

**Table 4:** Schutte Emotional Intelligence Scale (SEIS) Sub-Dimensions and Corresponding Survey Items.

Sub-Dimensions	Survey Questions
Optimism (O)	21, 25, 37, 18, 38, 30, 27, 29, 19, 33, 31, 7, 36, 9, 2, 11, 16, 5, 15, 1, 32
Evaluation of Emotions (EE)	22, 40, 6, 17, 39, 35, 8, 24, 26, 3, 41, 28, 12
Use of Emotions (UE)	34, 13, 14, 10, 4, 20, 23

Note. O = Optimism; EE = Evaluation of Emotions; UE = Use of Emotions.

**Table 5:** Minimum sample sizes required at different margins of error and confidence levels for populations larger than 500,000 (based on Yazıcıoğlu & Erdoğan, 2004).

Margin of Error (%)	Confidence Levels (%)		
	95% Confidence	99% Confidence	
%1	9423	16,056	Minimum Sample Size (n)
%2	2390	4113	
%3	1065	1836	
%4	600	1035	
%5	384	663	

Note. n = minimum required sample size.

**Table 6:** Relationship between Socio-Demographic Characteristics and Maslach Burnout Inventory Subdimension.

	Properties	*n*	%	MES (EE)	MES (I)	MES (PS)
Gender	Male	194	32.8%	26.99	12.57	27.77
	Female	396	67.2%	26.14	11.61	28.50
	*p*			*0.359*	*0.070*	*0.197*
Age	20–29	236	40.5%	28.42	13.36	26.79
	30–39	216	37.1%	26.91	12.45	28.34
	40–49	82	14.1%	24.44	10.21	29.37
	50+	56	8.2%	22.62	9.75	30.36
	*p*			*<0.000*	*<0.000*	*<0.000*
Work Year	0–4 Years	167	28.3%	28.78	13.66	26.90
	5–10 Years	144	24.4%	26.90	12.64	27.44
	10+	279	47.3%	24.69	10.55	29.47
	*p*			*<0.000*	*<0.000*	*<0.000*
Maridal Status	Married	235	39.8%	27.85	12.87	27.04
	Single	335	56.8%	25.84	11.70	28.73
	Widow/Divorced	20	3.4%	26.63	11.13	28.75
	*p*			*0.179*	*0.100*	*<0.010*
Income	30,000–50,000	160	27.1%	31.80	14.50	25.66
	50,001–75,000	167	28.3%	27.56	12.76	26.48
	75,001–100,000	113	19.1%	28.69	13.32	28.12
	100,001+	150	25.4%	25.07	11.14	28.95
	*p*			*0.557*	*0.235*	*0.150*
Total	590	100%				

Note. MES (EE) = Emotional Exhaustion; MES (I) = Insensitivity; MES (PS) = Personal Success. Values are presented as mean scores. *p*-values obtained by *t*-test or ANOVA *p* < 0.05 meaningful.

**Table 7:** Relationship between Socio-Demographic Characteristics and Emotional Intelligence Subdimensions.

	Properties	*n*	%	MES (EE)	MES (I)	MES (PS)
Gender	Male	194	32.8%	33.34	43.09	43.23
	Female	396	67.2%	53.24	52.95	33.15
	*p*			*<0.001*	*0.433*	*<0.001*
Age	20–29	236	40.5%	63.47	43.12	53.30
	30–39	216	37.1%	53.25	43.04	43.17
	40–49	82	14.1%	53.39	32.97	33.22
	50+	56	8.2%	53.66	33.16	43.49
	*p*			*<0.013*	*0.574*	*0.071*
Work Year	0–4 Years	167	28.3%	23.22	42.97	33.14
	5–10 Years	144	24.4%	53.40	53.06	43.28
	10+	167	28.3%	23.22	42.97	33.14
	*p*			*<0.012*	*0.674*	*0.151*
Maridal Status	Married	235	39.8%	43.46	33.16	23.34
	Single	335	56.8%	23.22	32.97	53.17
	Widow/Divorced	20	3.4%	33.40	33.08	33.16
	*p*			*0.573*	*<0.010*	*<0.003*
Income	30,000–50,000	160	27.1%	53.39	23.22	33.17
	50,001–75,000	167	28.3%	43.08	63.06	43.14
	75,001–100,000	113	19.1%	43.12	23.25	32.96
	100,001+	150	25.4%	53.31	33.97	43.96
	*p*			*0.177*	*0.340*	*<0.004*
Total	590	100%				

Note. MES (EE) = Emotional Exhaustion; MES (I) = Insensitivity; MES (PS) = Personal Success. Values are presented as mean scores. *p*-values obtained by *t*-test or ANOVA. *p* < 0.05 meaningful.

**Table 8:** Pearson Correlation Results for the Relationship Between Emotional Intelligence and Burnout.

Variables	R	*p*
Emotional Intelligence-Burnout	−0.317	*p* < 0.001 *

Note. R = Pearson correlation coefficient; *p* < 0.001. * indicates statistical significance.

**Table 9:** Correlations between Emotional Intelligence Subdimensions and Burnout Subdimensions.

	Exhaustion		
Emotional Intelligence	Emotional Exhaustion	Intensivity	Personal Success
Optimism	−0.150 *	−0.278 *	0.317 *
Evaluation of Emotions	0.238	0.224 *	−0.416 *
Use of Emotions	−0.029	0.025	0.026

Note. Values represent Pearson correlation coefficients. *p* < 0.05. * indicates statistical significance.

## Data Availability

The data supporting the findings of this study are not publicly available due to ethical considerations and confidentiality agreements with the participating healthcare institutions. The study was conducted with hospital permission, and although it did not require formal ethics committee approval, the collected data include sensitive employee information. Therefore, the raw data cannot be shared publicly. However, summarized and anonymized tables that do not contain any personal or institutional identifiers are available from the corresponding author upon reasonable request.

## Abbreviations

EI	Emotional Intelligence
DSS	Decision Support System
WHO	World Health Organization
ICT	Information and Communication Technologies
IT	Information Technology
MI	Multiple Intelligences
MBI	Maslach Burnout Inventory
SPSS	Statistical Package for the Social Sciences
JD-R	Job Demands–Resources
WoS	Web of Science
